# Efficient Connectivity in Smart Homes: Enhancing Living Comfort through IoT Infrastructure

**DOI:** 10.3390/s24092761

**Published:** 2024-04-26

**Authors:** Hamdy M. Youssef, Radwa Ahmed Osman, Alaa A. El-Bary

**Affiliations:** 1 Mechanical Engineering Department, College of Engineering and Architecture, Umm Al Qura University, Makkah 21955, Saudi Arabia; hmyoussef@uqu.edu.sa; 2 Basic and Applied Science, College of Engineering, Arab Academy for Science, Technology and Maritime Transport, Alexandria P.O. Box 1029, Egypt; aaelbary@aast.edu; 3 National Committee for Mathematics, Academy of Scientific Research and Technology, Cairo 4262104, Egypt; 4 Council of Future Studies and Risk Management, Academy of Scientific Research and and Technology, Cairo 4262104, Egypt

**Keywords:** smart home, Lagrange optimization, 1-DCNN, energy efficiency, achievable data rate

## Abstract

Modern homes are experiencing unprecedented levels of convenience because of the proliferation of smart devices. In order to improve communication between smart home devices, this paper presents a novel approach that particularly addresses interference caused by different transmission systems. The core of the suggested framework is an intelligent Internet of Things (IoT) system designed to reduce interference. By using adaptive communication protocols and sophisticated interference management algorithms, the framework minimizes interference caused by overlapping transmissions and guarantees effective data sharing. This can be accomplished by creating an optimization model that takes into account the dynamic nature of the smart home environment and intelligently allocates resources. By maximizing the signal quality at the destination and optimizing the distribution of frequency channels and transmission power levels, the model seeks to minimize interference. A deep learning technique is used to augment the optimization model by adaptively learning and predicting interference patterns from real-time observations and historical data. The experimental results show how effective the suggested hybrid strategy is. While the deep learning model adjusts to shifting interference dynamics, the optimization model efficiently controls resource allocation, leading to better data reception performance at the destination. The system’s robustness is assessed in various kinds of situations to demonstrate its flexibility in responding to changing smart home settings. This work not only offers a thorough framework for interference reduction but also clarifies how deep learning and mathematical optimization can work together to improve the dependability of data reception in smart homes.

## 1. Introduction

The Internet of Everything has arrived with the rapid development of Internet of Things (IoT) technology in recent years. A number of applications have emerged, including smart manufacturing, smart homes, automatic driving, health monitoring, smart agriculture, and smart metres [1,2]. The IoT is the engine powering improved network monitoring and control, from intelligent energy metres to the placement of sensors at strategic sites from production facilities to distribution hubs. Further improvements are needed in mobile network ecological structure and resource management to fulfil the demands of big machine-type communications in the IoT and to give users of the network a satisfying service experience [3].

It has become common for smart houses to come equipped with integrated communication systems. In order to meet the ideal home-energy profile and maintain a pleasant lifestyle, both grid operators and home users will soon be able to monitor and operate a number of household appliances. To create home area networks (HMAs), a variety of wired communication schemes, including power-line communication (PLC), inter-integrated circuit (I2C), and serial peripheral interface, as well as wireless technologies, including Wi-Fi, Zigbee, RFID, and the IoT, can be chosen based on the characteristics of the home [4]. Inherent flexibility and the capacity to move data between networks without a direct connection between users and computers have greatly aided in data analysis, task scheduling, device connections, and storage [5,6].

While IoT has several advantages, it also has a number of serious disadvantages, such as latency, security and privacy concerns, node or connection failures, communication protocols, and network efficiency [7]. These issues can all have a detrimental effect on the overall performance of the network. While security and privacy are the most frequently discussed constraints of IoT systems in the literature [8], interference mitigation is one of the challenges that still require further research. Blockchain technology, artificial intelligence (AI), and machine learning (ML) are further techniques being researched to enhance the functionality of IoT systems [9]. Furthermore, the fifth generation (5G) network is a powerful IoT strategy capable of supporting hundreds of medical devices. Several 5G-enabled IoT techniques have been presented [10,11] to support IoMT devices. Optimization techniques are essential for enhancing system performance and producing diverse model solutions in a multitude of domains and aspects, apart from machine learning and artificial intelligence [12,13].

IoT devices employ the 5G spectrum, a vital medium shared with other cellular user equipment (CUE) and other devices, to send data to gateways or other specified destinations. The fundamental difficulty is the possibility of interference at these gateways or destinations, which might harm the dependability and effectiveness of IoT connectivity. This interference is a serious problem, especially for vital applications like healthcare, where patient safety is directly impacted by data transmission accuracy. This work presents a novel approach that combines a mathematical optimization model and a deep learning algorithm to tackle these problems in a complete way. By working together, the interference problems that IoT devices face while using shared spectrum should be tackled. The suggested approach aims to improve the network’s reliability and efficiency by concentrating on the optimization of IoT communication strategies designed for smart homes, thereby guaranteeing dependable and smooth data transmission. The proposed approach does more than just address interference; it also makes smart home networks better overall. The suggested framework lays the groundwork for more durable and dependable smart home environments by placing a higher priority on the accuracy of collected data. In brief, the key contributions of this paper are as follows:

- In order to facilitate efficient and precise information delivery from smart home devices to their destinations, the proposed strategy developed a novel technique for controlling interference among IoT device connections. The Lagrange optimization technique was used to design an optimization problem with the installation of 1 D-CNN in order to ascertain the reliability of communication between home gateways and their destinations.
- The recommended approach aims to improve IoT network connections, especially for smart home devices that are experiencing extreme circumstances. This can be achieved by determining the necessary interference distance between the smart home gateways and any interfered distance to convey dependable data in various environmental circumstances from their intended destinations. On the other hand, path loss, the necessary signal-to-interference-plus-noise ratio ($SINR_{th}$), transmission power, and the presence of various interfering devices are other variables that could affect system performance.
- In order to receive precise and trustworthy data, home gateways will be able to predict, based on the channel circumstances, the maximum appropriate interference distance to reach accurate data by means of a deep learning model.
- The proposed method’s overall achievable data rate and energy efficiency were analyzed under a variety of environmental conditions, such as transmission power, required ($SINR_{th}$) values, and different transmission ranges. These results make it possible to optimize IoT networks for smart homes.

The following sections are arranged in the following order: The work on IoT will be presented in Section 2. The details of the recommended approach will be covered in Section 3. In Section 4, the analytical and experimental work for the proposed approach will be given. The work suggested in this paper will be concluded in Section 5.

## 2. Related Work

Many studies have been conducted on interference management and control in general, but not many have looked into this specifically in the context of smart homes. In order to address user-system conflicts in smart homes, Ref. [14] proposed a definition and mechanism for detecting them. It sought to increase user satisfaction and provide developers with guidance for building more dependable smart home systems based on an empirical study involving 163 users. RF sensing holds promise for improving healthcare and getting to know inhabitants as smart homes develop, but it also causes wireless congestion. In order to improve functionality, Ref. [15] suggested a collaborative design strategy that enhanced WiFi signals and integrated communication with sensing equipment. The aim was pervasive connectivity and inconspicuous sensing for smooth smart home operation. Additionally, Ref. [16] created a reference architecture with approachable security procedures for the IoT, which was the goal of this paper. It presented the first comprehensive strategy for smart-home activity, the traffic monitoring mechanism NDFA. The GHOST-IoT dataset is produced by NDFA and records actual IoT network traffic. With this dataset, NDFA’s capability to handle unprocessed network traffic from various interfaces and IoT in an actual smart home is demonstrated.

Furthermore, Ref. [17] optimized video streaming with wireless micro medical devices (WMMDs) in smart healthcare homes through the use of AI and IoT. By using a video transmission rate control algorithm (VTRCA) and a lazy video transmission algorithm (LVTA), it was able to stream videos through WMMD with notable energy savings and increased performance over the baseline. The growing risk of hacking assaults on smart building systems was discussed in [18]. The approach focused on locally identifying abnormalities and highlights how crucial it is for network providers to identify and correlate anomalies on a wider scale. By utilising machine learning, the method recognised questionable activities at the data centre of the service provider, encouraging shared accountability for the security of smart homes. Additionally, Ref. [19] also provided firewalling for IoT traffic. This technique, which is well known in computer networks, enables traffic routed to addresses that these end devices have never used before to be detected. As a result, after warning authorities, undesired traffic from smart devices may be banned, and the suspect device may be placed in quarantine.

Ref. [20] presented SEED, a novel and secure method for collecting data from IoT devices, in terms of energy efficiency for IoT networks. Compared to existing methods, SEED offered improved throughput and energy efficiency by using MD5 hashing to assure data integrity and upgrading aggregator nodes to address network issues. Additionally, Ref. [21] provided an energy-efficient Massive MIMO-NOMA IoT network for communications beyond 5G and addressed concerns with fast data transfer. By utilising fractional programming and sequential convex approximation, the proposed method outperformed previous methods in terms of energy efficiency, convergence, and user fairness. Furthermore, Ref. [22] suggested an interference control strategy to improve 5G cellular networks and IoT. Lagrange optimization was used to reduce interference, which improved system reliability and energy efficiency—two crucial QoS criteria. The simulation’s results demonstrated considerable improvements in network performance. In addition, the implementation of the dynamic Frequent Frequency Reuse (FFR) strategy, which was presented in [23], for maximizing network capacity by decreasing interference in the 5G system is one of these techniques. To mitigate interference concerns during the cohabitation of 5G and IoT networks, Ref. [24] introduced a distributed deep learning model designed to avoid interference. By predicting optimal distances for various communication circumstances, the model beat the state-of-the-art benchmarks in terms of throughput, energy efficiency, and interference suppression. Furthermore, Ref. [25] described a strategy for improving IoE network performance using Lagrange optimization and deep learning. It optimized transmission power for efficiency and throughput while minimising interference. A distributed deep learning network predicted optimal transmission power using Lagrange optimization data, which was validated by testing.

This study explores a crucial component of smart house infrastructure in order to improve the communication dependability between smart home devices and their destinations. Finding the ideal interference distance between house gateways—the central hubs gathering data for IoT smart homes and any interfered devices—in situations where other transmitting devices sharing the same frequency band could cause interference is the main goal. The goal is to pinpoint critical elements and setups that will increase smart home connectivity via enhanced IoT networks. The suggested methodology incorporates both an analytical optimization strategy and a deep learning model to address this difficulty. The method optimizes communication in smart homes by teaching IoT devices to dynamically alter their proximity to the targeted destinations by utilising a distributed deep learning model for IoT networks.

## 3. Materials and Methods

This section describes the suggested approach approach for enhancing communication between smart home devices and their intended destinations. In the suggested model, Lagrange optimization and 1D-CNN approaches were used to determine the ideal interference distance for improving IoT connectivity in smart home situations. Lagrange optimization is implemented as a mathematical optimization technique to maximize the performance of IoT communication systems while adhering to restrictions such as transmission distance, signal-to-interference-plus-noise ratio (SINR), and transmission power. Through Lagrange optimization, we were able to derive an analytical solution that best balances the trade-offs between interference mitigation and communication dependability by transforming the problem into a limited optimization assignment. In addition to Lagrange optimization, a 1D-CNN architecture is used to improve the accuracy of interference distance prediction. The 1D-CNN model was trained using simulated or real-world data to discover complicated patterns and correlations between input features (such as interference levels, signal strength, and environmental parameters) and the ideal interference distance. By employing deep learning capabilities, the 1D-CNN model allowed us to capture nonlinear correlations and make accurate predictions, improving the effectiveness of interference management tactics in smart home environments. The combination of Lagrange optimization with 1D-CNN enabled us to create a reliable and data-driven method for identifying the optimal interference distance to boost IoT connectivity in smart homes. Our research approach, which integrates mathematical optimization concepts with machine learning approaches, provides a complete and systematic framework for addressing interference management difficulties in complicated wireless communication systems.

### 3.1. System Model and Problem Formulation

**Figure 1:**
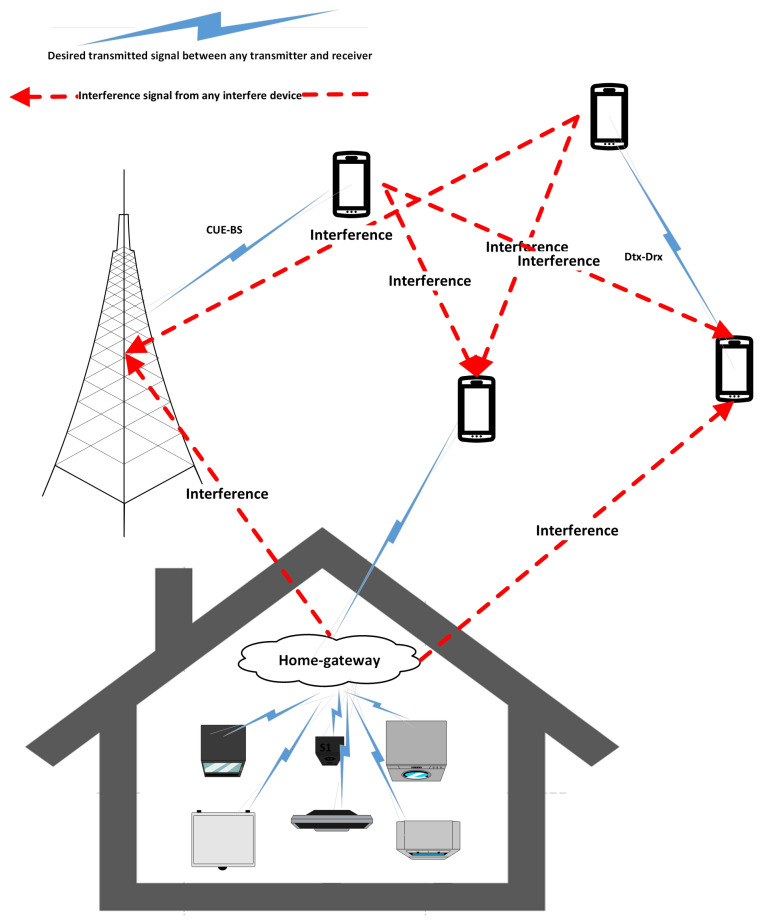
Proposed smart home communication system model.

For the proposed IoT network for smart homes, it is assumed that there are multiple appliances which directly communicate with the home gateways (*G*) and these home gateways have to send data to their desired destinations. A base station (BS) communicates with *C* cellular user equipment (CUEs), and *D* D2D communication pairs, consisting of transmitting devices (Dtx) and receiving devices (Drx), share the spectrum, as illustrated in Figure 1. Communication scenarios include: (i) sending data from any smart home’s appliances to the home gateways, which are subsequently in charge of forwarding the data to the intended location; (ii) standard cellular communication, in which CUEs communicate with BS; and (iii) Dtx and Drx communicating D2D. Interference is introduced to the destination if at least one CUE, or transmitting device (Dtx), sharing the same spectrum as home gateways, has data to broadcast to BS or any receiving devices (Drx), respectively. Thus, by reducing interference, the suggested methodology aims to improve communication between home gateways and their destinations. The goal is to maximize the smart home IoT network’s overall performance while taking into consideration the following equations, which represent the total attainable data rate (*R*) and energy efficiency (EE):

(1) $$\begin{array}{cc} \mathrm{Maximize}\; & \sum_{g=1}^{G}\sum_{c=1}^{C}\sum_{d=1}^{D}{EE}_{g,c,d}\\ \mathrm{Subject}\;\mathrm{to}\; & {EE}_{g,c,d}:=f_{1}\left(SINR_{GD},P_{C},P_{D}\right)\\ \; & \left\{SINR_{GD}\geq SINR_{th},\;P_{C}\leq P_{Cmax},\;P_{D}\leq P_{Dmax}\right\} \end{array}$$

(2) $$\begin{array}{cc} \mathrm{Maximize}\; & \sum_{g=1}^{G}\sum_{c=1}^{C}\sum_{d=1}^{D}R_{g,c,d}\\ \mathrm{Subject}\;\mathrm{to}\; & R_{g,c,d}:=f_{2}\left(SINR_{GD},P_{C},P_{D}\right)\\ \; & \left\{SINR_{GD}\geq SINR_{th},\;P_{C}\leq P_{Cmax},\;P_{D}\leq P_{Dmax}\right\} \end{array}$$

Thus, in the context of the optimization problem, the system energy efficiency is denoted by $EE_{g,c,d}$ and the total possible data rate is denoted by $R_{g,c,d}$. These measures are related to the *d*-th road between D2D devices, the *k*-th path between CUE and BS, and the *i*-th path between home gateways and their intended destination. The symbols $SINR_{th}$ and $SINR_{GD}$ represent the required system signal-to-interference-plus-noise ratio and the signal-to-interference-plus-noise ratio for home gateways to any destination connection, respectively. Similarly, $P_{D}$ and $P_{Dmax}$ indicate the transmission power and maximum transmission power of the D2D communication connection, whereas $P_{C}$ and $P_{Cmax}$ represent the transmission power of the CUE and its maximum transmission power.

Non-orthogonal multiple access (NOMA) is chosen as the appropriate access method in the proposed paradigm [26,27] in order to enable concurrent access to the channel and to enable the wide implementation of IoT, CUE, and D2D. Additionally, the suggested model functions on the presumption of an additive white Gaussian noise (AWGN) Rayleigh fading channel [28]. Moreover, the model assumes statistical independence between the channel fading coefficients for various transmission connections. Consequently, the network’s achievable data rate (*R*) and energy efficiency (EE) can be expressed as follows:

(3) $$\begin{array}{c} EE=\frac{R_{GD}}{P_{G}+P_{o}}+\frac{R_{CB}}{P_{C}+P_{o}}+\frac{R_{DD_{rx}}}{P_{D}+P_{o}} \end{array}$$

(4) $$\begin{array}{c} R=R_{GD}+R_{CB}+R_{DD_{rx}} \end{array}$$

where the achievable data rates for the home gateways and their destinations link, CUE-BS link, and D2D communication link are represented by the symbols $R_{GD}$, $R_{CB}$, and $R_{DD_{rx}}$, respectively. The values $P_{G}$ and $P_{o}$, respectively, indicate the internal circuit power consumption and home gateway transmission power for chronic patients. As a result, the following are the expressions for $R_{GD}$, $R_{CB}$, and $R_{DD_{rx}}$:

(5) $$\begin{array}{c} R_{GD}=B\log_{2}\left(1+\frac{P_{G}H_{GD}}{\sum_{k=1}^{K}P_{C}H_{C_{k}D}+\sum_{d=1}^{D}P_{D}H_{D_{d}D}+N}\right) \end{array}$$

(6) $$\begin{array}{c} R_{CB}=B\log_{2}\left(1+\frac{P_{C}H_{CB}}{\sum_{g=1}^{G}P_{G}H_{G_{g}B}+\sum_{d=1}^{D}P_{D}H_{D_{d}B}+N}\right) \end{array}$$

(7) $$\begin{array}{c} R_{DD_{rx}}=B\log_{2}\left(1+\frac{P_{D}H_{DD_{rx}}}{\sum_{g=1}^{G}P_{G}H_{G_{g}D_{rx}}+\sum_{k=1}^{K}P_{C}H_{C_{k}D_{rx}}+N}\right) \end{array}$$

where the channel gain coefficients between the home gateways and the intended destination, CUE and BS, Dtx and Drx, respectively, are denoted by the symbols $H_{GD}$, $H_{C_{k}D}$, and $H_{D_{d}D}$. The channel gain coefficients between CUE and BS, home gateways and BS, and Dtx and BS are denoted by the variables $H_{CB}$, $H_{G_{g}B}$, and $H_{D_{d}B}$, respectively. The channel gain coefficients between Dtx and Drx, home gateways and Dtx, and CUE and Drx are, respectively, $H_{DD_{rx}}$, $H_{G_{g}D_{rx}}$, and $H_{C_{k}D_{rx}}$. The channel gains are measured in dB. Additionally, in this case, *N* and *B* represent the noise power and channel system bandwidth which are measured in dBm and hertz, respectively.

The principal aim of the suggested methodology is to maximize the achievable data rate (*R*) and overall energy efficiency (EE) in diverse environmental scenarios, as indicated by Equations (1) and (2). Thus, Equations (1) and (2) reflect the optimization problem, and the Lagrangian for them is as follows:

(8) $$\begin{array}{c} L\left({SINR}_{GD},P_{C},P_{D},\lambda_{1},\lambda_{2},\lambda_{3}\right)=EE+\lambda_{1}\left({SINR}_{GD}-{SINR}_{th}\right)\\ +\lambda_{2}\left(P_{C}-P_{Cmax}\right)++\lambda_{3}\left(P_{D}-P_{Dmax}\right). \end{array}$$

(9) $$\begin{array}{c} L\left({SINR}_{GD},P_{C},P_{D},\mu_{1},\mu_{2},\mu_{3}\right)=R+\mu_{1}\left({SINR}_{ID}-{SINR}_{th}\right)\\ +\mu_{2}\left(P_{C}-P_{Cmax}\right)+\mu_{3}\left(P_{D}-P_{Dmax}\right). \end{array}$$

The non-negative Lagrangian multipliers in this instance are $\lambda_{1}$, $\lambda_{2}$, $\lambda_{3}$, $\mu_{1}$, $\mu_{2}$, and $\mu_{3}$. The values of $\lambda_{1}$, $\lambda_{2}$, and $\lambda_{3}$ must be ascertained by considering the derivative of Equation (8) with reference to $P_{G}$, $P_{C}$, and $P_{D}$ in order to fulfil the requirements of the energy efficiency (EE) optimization issue. Consequently, the following can be used to obtain $\lambda_{1}$, $\lambda_{2}$, and $\lambda_{3}$:

(10) $$\begin{array}{ccc} \lambda_{1} & =\frac{B\cdot X_{1}}{\left(P_{G}+P_{o}\right)}+\frac{B\cdot \log_{2}\left(1+P_{G}\cdot X_{2}\right)}{{\left(P_{G}+P_{o}\right)}^{2}\cdot X_{2}} & \;+\frac{B\cdot X_{3}\cdot X_{4}}{\left(P_{C}+P_{o}\right)\cdot X_{2}}+\frac{B\cdot X_{5}\cdot X_{6}}{\left(P_{D}+P_{o}\right)\cdot X_{2}} \end{array}$$

(11) $$\begin{array}{cc} \lambda_{2} & =\frac{B\cdot X_{1}}{\left(P_{G}+P_{o}\right)}\cdot \frac{P_{G}\sum_{k=1}^{K}H_{c_{k}D}\cdot X_{2}}{C}+\frac{B\cdot \log_{2}\left(1+P_{C}\cdot X_{7}\right)}{{\left(P_{C}+P_{o}\right)}^{2}}-\frac{B\cdot X_{3}\cdot X_{7}}{\left(P_{C}+P_{o}\right)}\\ & \;+\frac{B\cdot X_{5}\cdot X_{8}}{\left(P_{D}+P_{o}\right)}+\lambda_{1}\left(\frac{P_{G}\sum_{k=1}^{K}H_{C_{k}D}\cdot X_{2}}{C}\right) \end{array}$$

(12) $$\begin{array}{cc} \lambda_{3} & =\frac{B\cdot X_{1}}{\left(P_{G}+P_{o}\right)}\cdot \frac{P_{G}\sum_{l=1}^{L}H_{D_{d}D}\cdot X_{2}}{C}+\frac{B\cdot X_{3}\cdot X_{9}}{\left(P_{C}+P_{o}\right)}+\frac{B\cdot \log_{2}\left(1+P_{D}X_{10}\right)}{{\left(P_{D}+P_{o}\right)}^{2}}\\ & \;-\frac{B\cdot X_{5}\cdot X_{10}}{\left(P_{D}+P_{o}\right)}+\lambda_{1}\left(\frac{P_{G}\sum_{d=1}^{D}H_{D_{d}D}\cdot X_{2}}{C}\right) \end{array}$$

where

$$\begin{array}{cc} C & =\sum_{k=1}^{K}P_{C}H_{C_{k}D}+\sum_{d=1}^{D}P_{D}H_{D_{d}D}+N, \end{array}$$

$$\begin{array}{cc} X_{1} & =\frac{\sum_{k=1}^{K}P_{C}H_{C_{k}D}+\sum_{d=1}^{D}P_{D}H_{D_{d}D}+N}{\sum_{k=1}^{K}P_{C}H_{C_{k}D}+\sum_{d=1}^{D}P_{D}H_{D_{d}D}+N+P_{G}H_{GD}},\\ X_{2} & =\frac{H_{GD}}{\sum_{k=1}^{K}P_{C}H_{C_{k}D}+\sum_{d=1}^{D}P_{D}H_{D_{d}D}+N},\\ X_{3} & =\frac{\sum_{g=1}^{G}P_{G}H_{G_{g}B}+\sum_{d=1}^{D}P_{D}H_{D_{d}B}+N}{\sum_{i=1}^{I}P_{I}H_{I_{i}B}+\sum_{d=1}^{D}P_{D}H_{D_{d}B}+N+P_{C}H_{CB}},\\ X_{4} & =\frac{P_{C}H_{CB}\sum_{g=1}^{G}H_{G_{g}B}}{{\left(\sum_{g=1}^{G}P_{G}H_{G_{g}B}+\sum_{d=1}^{D}P_{D}H_{D_{d}B}+N\right)}^{2}},\\ X_{5} & =\frac{\sum_{g=1}^{G}P_{G}H_{G_{g}D_{rx}}+\sum_{k=1}^{K}P_{C}H_{C_{k}D_{rx}}+N}{\sum_{g=1}^{G}P_{G}H_{G_{g}D_{rx}}+\sum_{k=1}^{K}P_{C}H_{C_{k}D_{rx}}+N+P_{D}H_{DD_{rx}}},\\ X_{6} & =\frac{P_{D}H_{DD_{rx}}\sum_{g=1}^{G}H_{G_{g}D_{rx}}}{{\left(\sum_{g=1}^{G}P_{I}H_{G_{g}D_{rx}}+\sum_{k=1}^{K}P_{C}H_{C_{k}D_{rx}}+N\right)}^{2}},\\ X_{7} & =\frac{H_{CB}}{\sum_{g=1}^{G}P_{G}H_{G_{g}B}+\sum_{d=1}^{D}P_{D}H_{D_{d}B}+N},\\ X_{8} & =\frac{P_{D}H_{DD_{rx}}\sum_{k=1}^{K}H_{C_{k}D_{rx}}}{{\left(\sum_{g=1}^{G}P_{G}H_{G_{g}D_{rx}}+\sum_{k=1}^{K}P_{C}H_{C_{k}D_{rx}}+N\right)}^{2}},\\ X_{9} & =\frac{P_{B}H_{CB}\sum_{d=1}^{D}P_{D}H_{D_{d}B}}{{\left(\sum_{g=1}^{G}P_{G}H_{G_{g}B}+\sum_{d=1}^{D}P_{D}H_{D_{d}B}+N\right)}^{2}}, \end{array}$$

$$\begin{array}{cc} X_{10} & =\frac{H_{DD_{rx}}}{\sum_{g=1}^{G}P_{G}H_{G_{g}D_{rx}}+\sum_{k=1}^{K}P_{C}H_{C_{k}D_{rx}}+N} \end{array}$$

Equations (10)–(12) are determined using Lagrange optimization techniques. Lagrange optimization is a strong mathematical method for solving restricted optimization problems that incorporates restrictions into the goal function using Lagrange multipliers. In this work, Lagrange optimization is used to maximize the system’s energy efficiency (EE) while respecting system restrictions such as transmission power, transmission lengths, path loss, and SINR. Equations (10)–(12) are the Lagrangian functions designed to maximize the objective function (energy efficiency) while adhering to the stipulated limitations. The Lagrange multipliers associated with these equations play an important role in defining the appropriate interference distance, ensuring that smart home communication systems operate efficiently.

Additionally, by deriving Equation (8) with respect to $\lambda_{1}$, $\lambda_{2}$, and $\lambda_{3}$, it is possible to find the optimal required interference distance ($d_{int}$) between the required destination for the data sent from the home gateways and any interfering devices, the optimal required CUE interference transmission power ($P_{C}$), and the optimal required Dtx interference transmission power ($P_{D}$).

(13) $$\begin{array}{c} d_{int}={\left[\frac{P_{G}H_{G_{D}}-N\cdot {SINR}_{th}}{{SINR}_{th}\left(\sum_{k=1}^{K}P_{C}pl_{o}+\sum_{d=1}^{D}P_{D}pl_{o}\right)}\right]}^{-1/\alpha } \end{array}$$

where the path loss exponent is α and the constant path loss is $pl_{o}$.

(14) $$\begin{array}{c} P_{C}=P_{C_{max}} \end{array}$$

(15) $$\begin{array}{c} P_{D}=P_{D_{max}} \end{array}$$

The values of $\mu_{1}$, $\mu_{2}$, and $\mu_{3}$ can be found using the derivative of Equation (9) with regard to $P_{I}$, $P_{C}$, and $P_{D}$ in order to satisfy the constraint of the optimization problem for (*R*). Then, we may represent $\mu_{1}$, $\mu_{2}$, and $\mu_{3}$ as follows:

(16) $$\begin{array}{c} \mu_{1}=\frac{B\cdot X_{1}\cdot X_{2}+B\cdot X_{3}\cdot X_{4}+B\cdot X_{5}\cdot X_{6}}{X_{2}} \end{array}$$

(17) $$\begin{array}{cc} \mu_{2} & =B\cdot X_{1}\left(\frac{P_{G}\sum_{k=1}^{K}H_{C_{k}D}\cdot X_{2}}{C}\right)-B\cdot X_{3}\cdot X_{7}+B\cdot X_{5}\cdot X_{8}\\ & \;+\lambda_{1}\left(\frac{P_{G}\sum_{k=1}^{K}H_{C_{k}D}\cdot X_{2}}{C}\right) \end{array}$$

(18) $$\begin{array}{cc} \mu_{3} & =BX_{1}\left(\frac{P_{G}\sum_{d=1}^{L}H_{D_{d}D}\cdot X_{2}}{C}\right)+B\cdot X_{3}\cdot X_{9}-B\cdot X_{5}\cdot X_{10}\\ & \;+\lambda_{1}\left(\frac{P_{G}\sum_{d=1}^{D}H_{D_{d}D}\cdot X_{2}}{C}\right) \end{array}$$

Equations (16)–(18) are developed using Lagrange optimization techniques to calculate the system’s maximum achievable data rate (R). Lagrange optimization is used to maximize data rate while adhering to restrictions like power allocation and interference management. Equations (16)–(18) are the Lagrangian functions designed to maximize the goal function (achievable data rate) under the given restrictions. The Lagrange multipliers associated with these equations are critical in calculating the appropriate power allocation method and interference distance, assuring the effective operation of smart home communication systems in obtaining the highest achievable data rate (R).

The optimal required interference distance ($d_{int}$) between home gateways and the required destination, the optimal required CUE interference transmission power ($P_{C}$), and the optimal required Dtx interference transmission power ($P_{D}$) can be obtained by deriving Equation (9) with respect to $mu_{1}$, $mu_{2}$, and $mu_{3}$. This will make it possible to optimize the overall attainable data rate (*R*), which may be calculated with the following formula:

(19) $$\begin{array}{c} d_{int}={\left[\frac{P_{G}H_{G_{D}}-N\cdot {SINR}_{th}}{{SINR}_{th}\left(\sum_{k=1}^{K}P_{C}pl_{o}+\sum_{d=1}^{D}P_{D}pl_{o}\right)}\right]}^{-1/\alpha } \end{array}$$

(20) $$\begin{array}{c} P_{C}=P_{C_{max}} \end{array}$$

(21) $$\begin{array}{c} P_{D}=P_{D_{max}} \end{array}$$

### 3.2. Dataset Generation

In this section, equations for the proposed model described in Section 3.1 have been implemented, and MATLAB (2018a) simulations have been used to provide the necessary datasets. The values of the simulation’s parameters are displayed in Table 1. The goal is to improve communication between home gateways and the intended destination by using the datasets to train models that will be installed on all transmitting devices.

**Figure 2:**
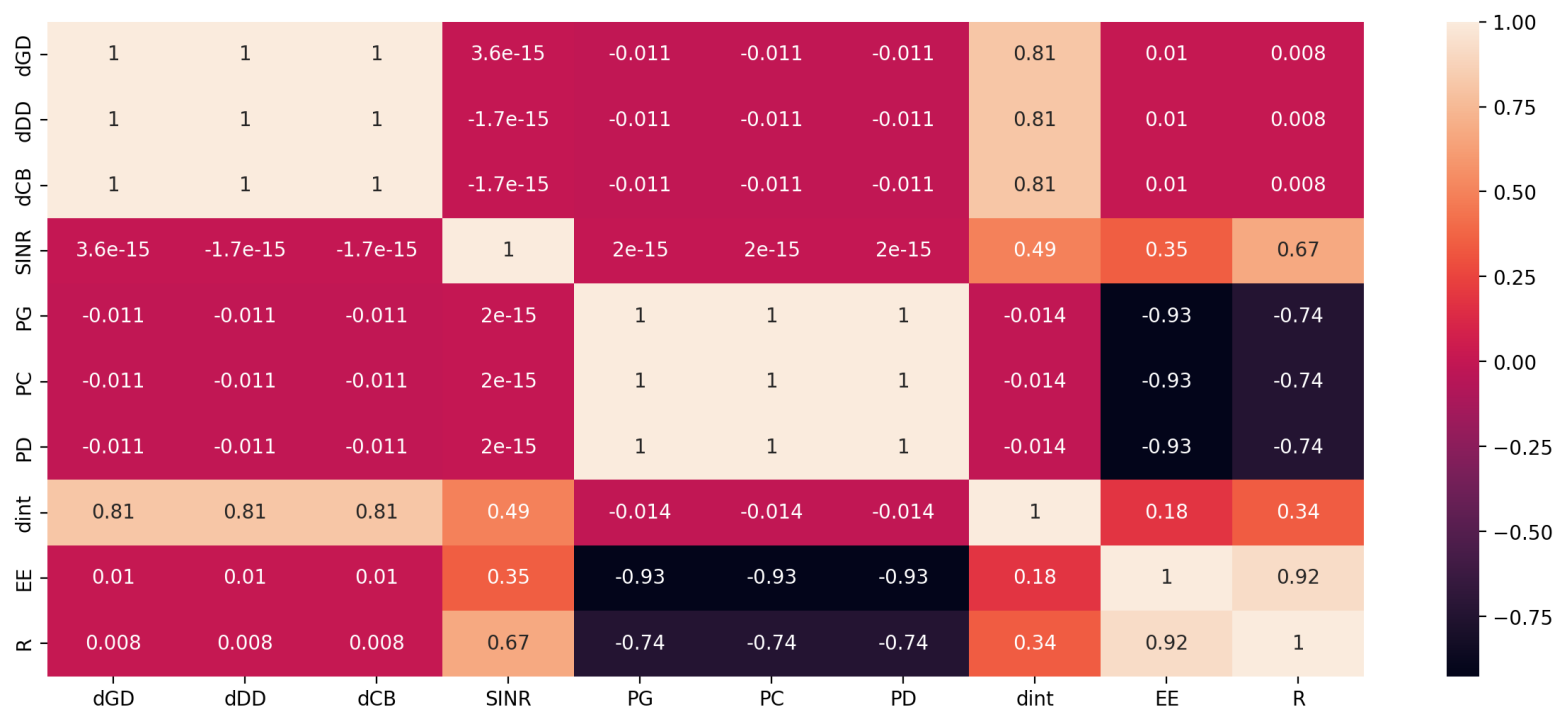
Pearson correlation coefficients of each input parameter ($d_{CB}$, $d_{DD_{rx}}$, $d_{CB}$, $SINR_{th}$, $P_{G}$, $P_{C}$, and $P_{D}$) and the output ($d_{int}$, EE and R).

There are 44,679 records in all. Each record contains a unique combination of these variables to represent the following: the distances ($d_{GD}$) between the home gateways and their destination, CUB and BS ($d_{CB}$), Dtx and Drx ($d_{DD_{rx}}$), the necessary signal-to-interference-plus-noise-ratio threshold ($SINR_{th}$), the home gateways ($P_{G}$), the CUE transmission power ($P_{C}$), and the D2D transmission power ($P_{D}$). Figure 2 shows the Pearson coefficients that illustrate the relationship between each input and output parameter. The graph shows that EE and R have a substantial negative correlation with the $P_{G}$, $P_{C}$, and $P_{D}$ parameters, but the output $d_{int}$ has a strong association with the $d_{GD}$, $d_{CB}$, and $d_{DD_{rx}}$ parameters. Furthermore, there is not much of a link between parameters *R* and the input parameters. Each of these variables must be used to train the deep learning model, and the results section will provide an explanation of the association’s significance.

### 3.3. Proposed Deep Learning Model

**Figure 3:**
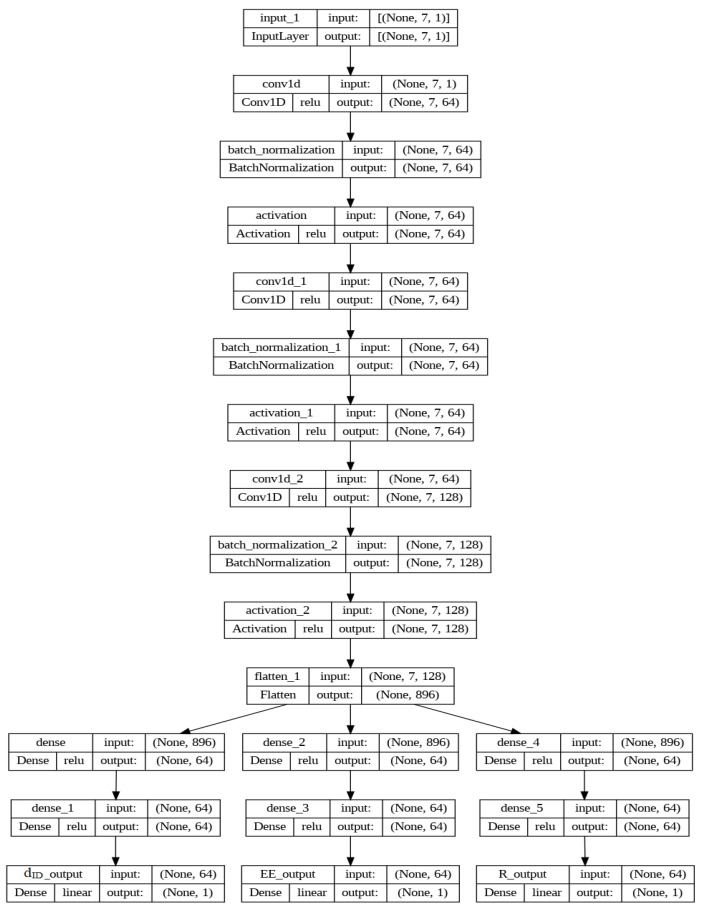
Proposed deep learning model.

In this section, the suggested deep learning model is demonstrated and explained. Before adding the variables to the recommended deep learning model, a normalization phase must be completed in order to help with the learning of the model weights. Each variable is normalized using the min-max scaling procedure before being incorporated into the model. Using the eight input variables, $d_{GD}$, $d_{DD_{rx}}$, $d_{CB}$, $SINR_{th}$, $P_{G}$, $P_{C}$, and $P_{D}$, the output parameters, $d_{int}$, EE, and *R*, are obtained from the final dense layer. The model has three distinct phases, namely 1D-CNN [32], flattening, and thick layers, as illustrated in Figure 3. The normalized input parameters are processed through three DCNN layers, as shown in Figure 3. The first layer has 64 filters and a kernel of size 1, the second has 64 filters and a kernel of size 1, and the third has 128 filters and a kernel of size one. Each 1D-CNN layer generates padded results to maintain the output matrix width. The output of the third 1D-CNN is then sent into a flattening layer, which restores the dimension and prepares it for input to the dense layers. Three dense layers follow the flattening layer to provide the regression result. A grid search was used to examine various combinations of the number of filters in the 1D-CNN and nodes in dense layers.

To maintain a constant width of the output matrix, each layer of the 1D-CNN produces padded results. Next, a flattening layer receives the output from the third 1D-CNN and reformats the dimension to prepare it for input into the dense layers. Regression is produced by six dense layers that come after the flattening layer. Before choosing how many nodes to utilize for the dense layers and how many filters to employ for the 1D-CNN, a grid search was utilized to test out a number of options. The activation function for each hidden layer has been the rectified linear unit (ReLU). The grid search took activation function selection into consideration and tested several methods for following the hidden layers in the proposed model. For optimal results, the output of each hidden layer was input into an activation function known as a parametric rectified linear unit, or PReLU.

The root mean square error (RMSE) and mean absolute error (MAE) loss function are the objectives of the adaptive moment (Adam) optimization used in the proposed model. Adam’s learning method allows him to acquire the required abilities. Whereas RMSE is the root square of the average of the squared disparities between real and anticipated values, MAE measures the average difference between the actual and expected values. They can be referred to as these:

$$\begin{array}{c} MAE=\frac{\sum_{j=1}^{n}\left|y_{j}-x_{j}\right|}{n} \end{array}$$

$$\begin{array}{c} RMSE=\sqrt{\frac{\sum_{j=1}^{n}{\left(y_{j}-x_{j}\right)}^{2}}{n}} \end{array}$$

If $x_{j}$ is the anticipated value, $y_{j}$ is the actual value, and *n* is the total number of data points that were recorded. The experiments that were conducted in order to develop, validate, and test the proposed model are covered in the section that follows.

## 4. Results

**Figure 4:**
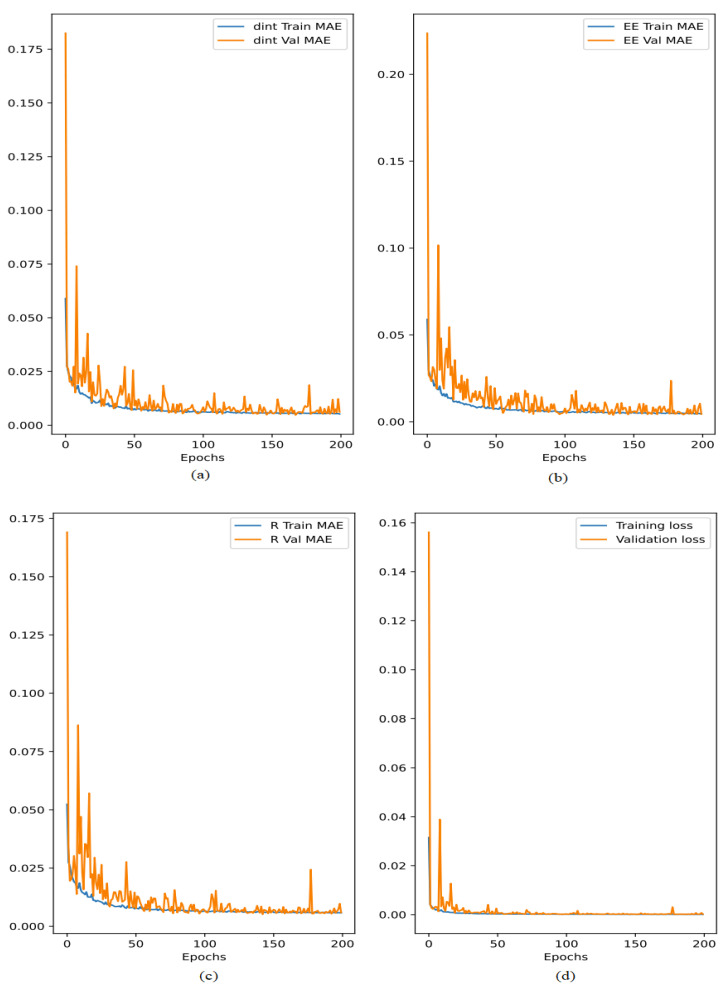
Training and validation mean absolute error generated during training the proposed model. (**a**) The training and validation mean absolute errors ($d_{int}$), (**b**) the training and validation mean absolute errors (EE), (**c**) the training and validation mean absolute errors (R) and (**d**) the training and validation mean absolute errors of the suggested model.

This section presents the performance of the suggested deep learning and analytical models. Furthermore, the effectiveness of the suggested method was assessed in terms of achievable data rate and improved energy efficiency using MATLAB and Python simulations. As seen in Figure 4, the suggested deep learning model from Section 3.3 is assessed and put to the test. An 80% train set and a 20% test set were created from the datasets. For the necessary $d_{int}$, EE, and *R*, respectively, the training and validation mean absolute errors are displayed in Figure 4a–c. Since the results were not changing noticeably beyond epoch 100, all of these graphs demonstrate that additional training was not necessary. Furthermore, each output in Figure 4d had about equal independent training and validation errors, indicating that the suggested model was neither overfit nor underfit. It also shows how the independent training and validation mistakes loss eventually decrease and stabilize.

**Figure 5:**
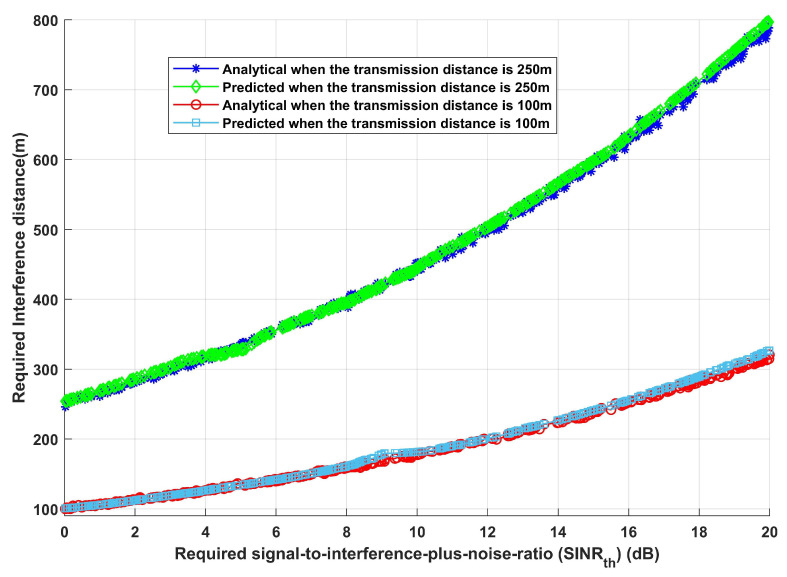
Required signal-to-interference-plus-noise ratio ($SINR_{th}$) versus required interference distance.

It has been assumed that the home gateway transmission power is always equal to the interference transmission power in order to show the efficacy and resilience of the proposed approach. Figure 5 illustrates the required linked signal-to-interference-plus-noise ratio ($SINR_{th}$) for both the analytical and deep learning methods, weighed against the necessary interference transmission distance between the home gateway and any interfered devices. There are two distinct transmission distances (100 and 250 m) that have been assumed in order to assess the effectiveness of the suggested model. Additionally, it has been assumed that every interfering device transmitted data using the 23 dBm maximum interference transmission power. In the worst scenario, data delivered from home gateways and their destinations can be impacted by significant amounts of interference transmission power. There is an optimal necessary interference transmission distance between home gateways and any interfering devices for each required $SINR_{th}$ for both the analytical and deep learning models in order to accomplish the required system ($SINR_{th}$). As seen in Figure 5, the ideal required interference distance for home gateways and any interfering devices for the analytical and deep learning models, respectively, to fulfil needed system ($SINR_{th}$), is 178.429 m and 181.6867 m when $SINR_{th}$ is 10 dB and transmission distance distance is 100 m. For the analytical and deep learning models, the optimal necessary interference distance is 452.5064 m and 445.61835 m, respectively, whereas the transmission distance is 250 m. By comparing the two cases, it is feasible to conclude that a certain interference distance between home gateways and any interfering equipment is needed to obtain the desired $SINR_{th}$. Moreover, it is noteworthy that increasing the required $SINR_{th}$ leads to a longer interference distance, which helps to reduce interference’s effects and attain the intended performance. This guarantees that the information will be received with sufficient accuracy and reliability and that it will be delivered via an effective communication route.

**Figure 6:**
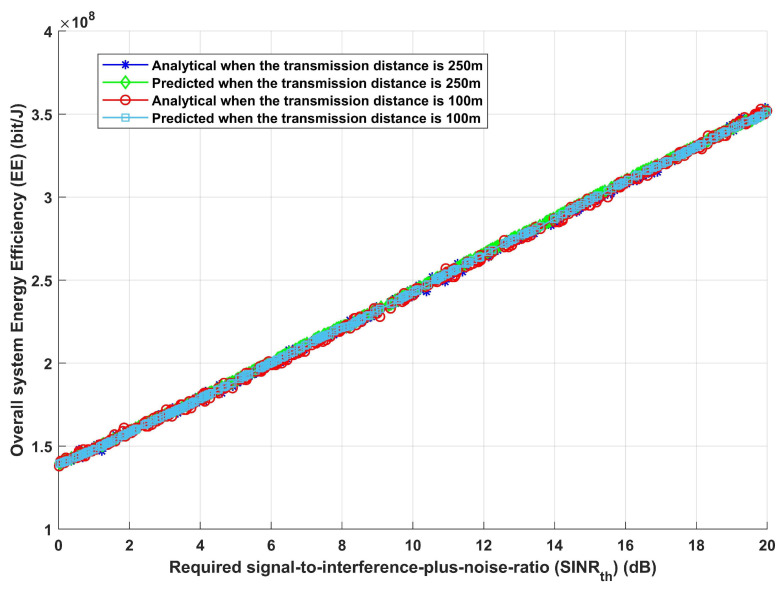
Required signal-to-interference-plus-noise ratio ($SINR_{th}$) versus overall system energy efficiency (EE).

**Figure 7:**
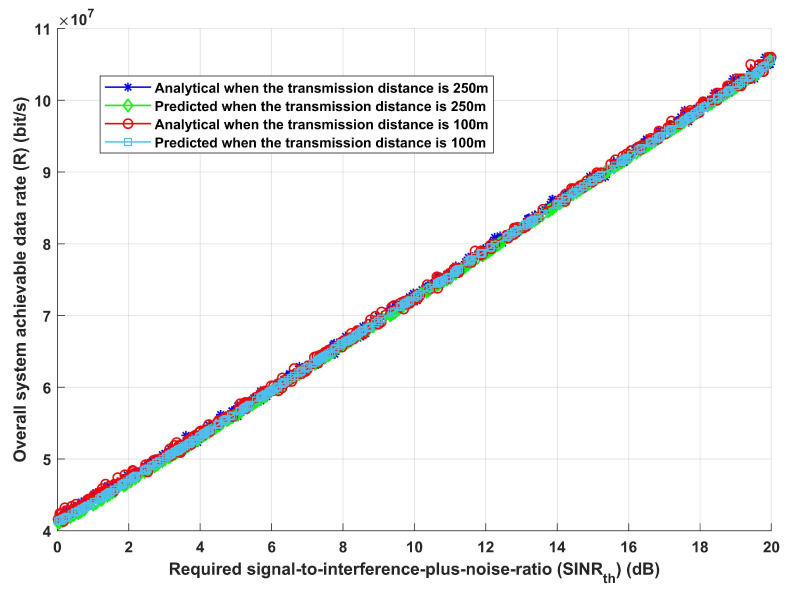
Required signal-to-interference-plus-noise ratio ($SINR_{th}$) versus overall system achievable data rate (R).

Furthermore, given the same previously stated assumed scenario, the system is assessed in terms of total system EE and overall achievable data rate, as shown in Figure 6 and Figure 7. Figure 6, which shows how EE increases with the increase in required $SINR_{th}$, for both analytical and deep learning models, demonstrates the effectiveness of the proposed model and its ability to modify the necessary interference distance between home gateways and any interfering devices in order to reach the maximum required EE. Furthermore, it can be observed that both the analytical and deep learning models perform equally well as the transmission distance rises. Moreover, the same result is obtained when assessing the system’s performance in terms of achievable data rate (R), as Figure 7 illustrates. Furthermore, increasing $SINR_{th}$ raises EE for both analytical and deep learning models, for both estimated transmission distances, as Figure 7 illustrates. This figure illustrates the effectiveness of the proposed model, which also validates the results from Figure 6. It can modify the interference distance based on the system’s needs to ensure dependable and efficient communication under a range of channel conditions.

It is noteworthy to emphasize that extra care should be taken in the strategic positioning of additional possibly interfering devices in circumstances where a range of IoT devices are responsible for data transmission. This is because Figure 5 provides insights into the discoveries made in Figure 6 and Figure 7. This becomes important when trying to properly stop, remove, or manage interference to obtain the required system performance. Maintaining rigorous control over the positioning of interfering equipment can boost the overall dependability of the data gathered and provide the foundation for essential emergency decision-making.

**Figure 8:**
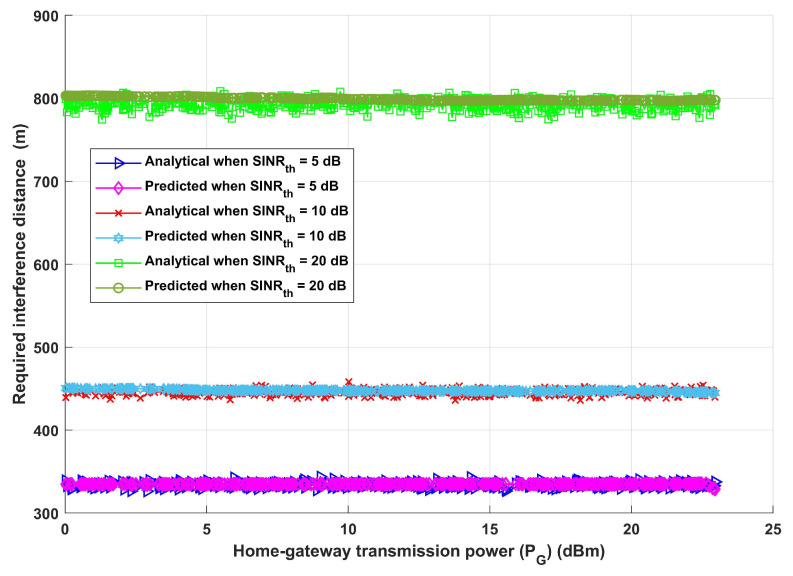
Home gateway transmission power ($P_{G}$) versus required interference distance.

In order to assess the system further, the required interference distance between the home gateway and any interfering devices is calculated versus home gateway transmission power ($P_{G}$), taking into account three distinct values for the ($SINR_{th}$) thresholds (5 dB, 10 dB, and 20 dB), and an interference transmission power equals to home gateway transmission power ($P_{G}$). Increasing $SINR_{th}$ produces an increase in the necessary interference distance between the home gateway and any interfering devices, as demonstrated in Figure 8. Due to the assumption that the interference power always equals $P_{G}$, it is noteworthy that increasing the home gateway transmission power does not change the required interference distance. Furthermore, it is vital to underline that an increase in the interference transmission distance is connected with increases in $SINR_{th}$. Put another way, as $SINR_{t}h$ increases, the system merely increases the interference distance to maintain efficient information exchange and boost overall dependability and efficiency under a variety of circumstances.

**Figure 9:**
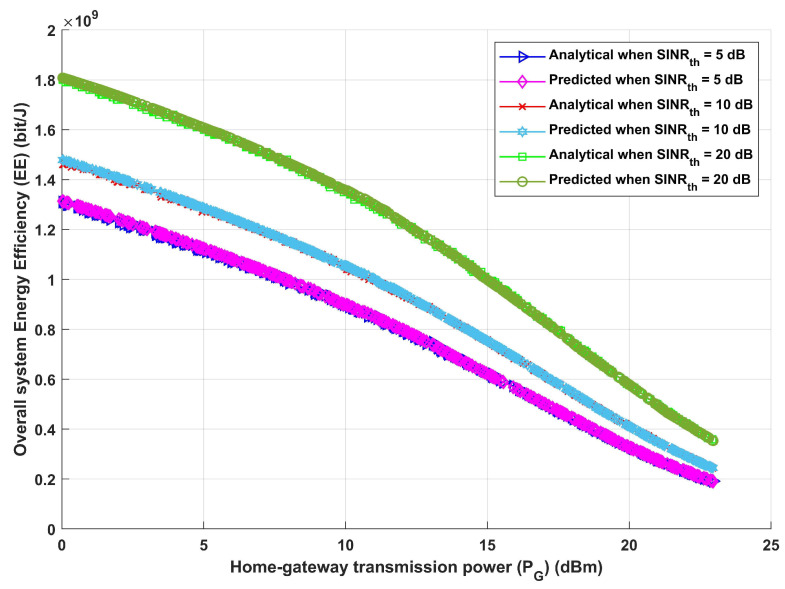
Home gateway transmission power ($P_{G}$) versus overall system energy efficiency (EE).

**Figure 10:**
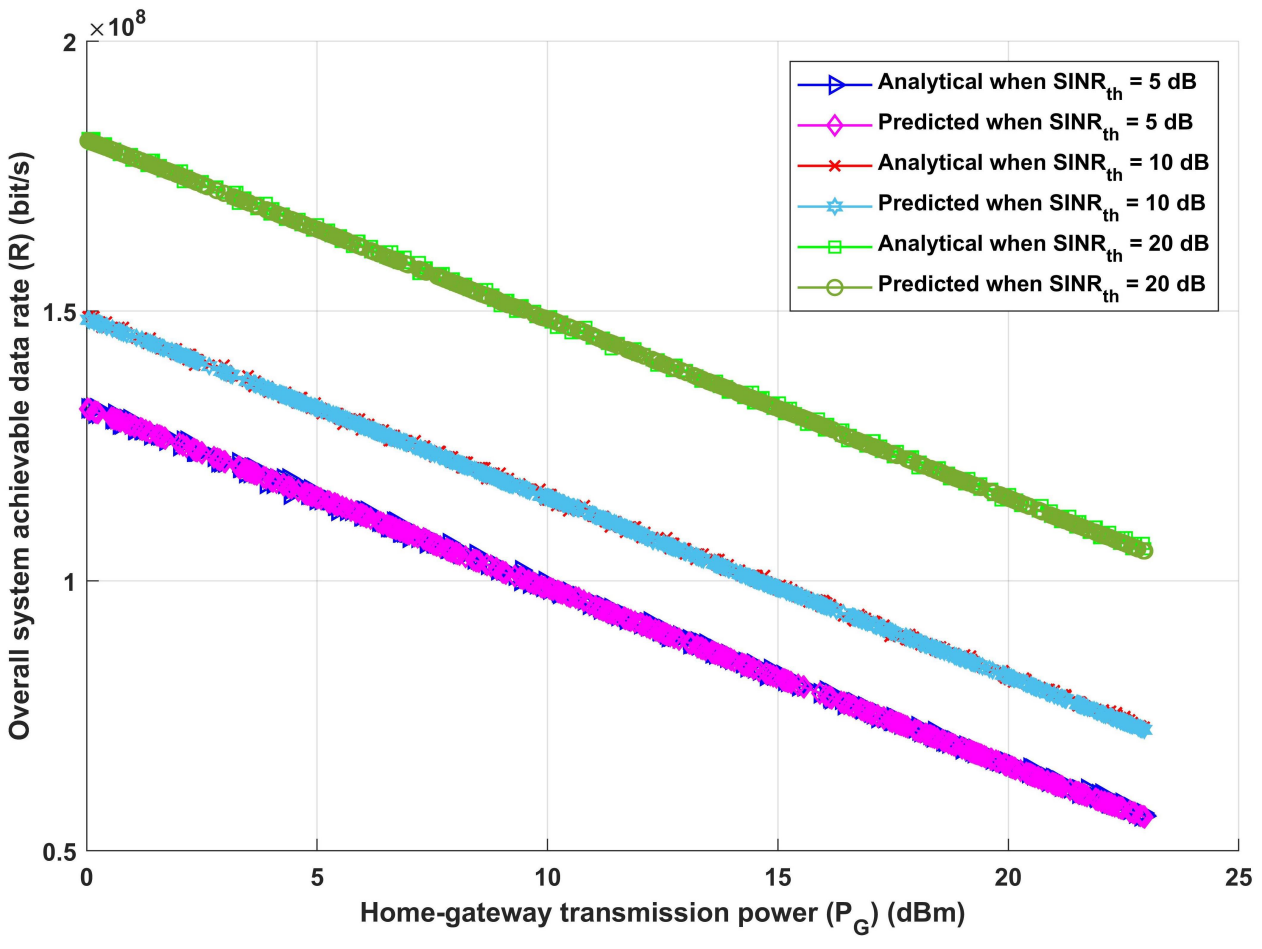
Home gateway transmission power ($P_{G}$) versus overall system achievable data rate (R).

Figure 9 and Figure 10 exhibit the correlation between home gateway transmission power ($P_{G}$) and the total energy efficiency of the system as well as the correlation between home gateway transmission power ($P_{G}$) and the total achievable data rate. As previously noted, three different values for $SINR_{th}$ have been taken into consideration. Using either the analytical or deep learning model, as the home gateway transmission power ($P_{G}$) increases, the overall system energy efficiency (EE) decreases, as shown in Figure 9. This is related to the growth in energy expenses accompanying greater power, paired with a matching increase in interference power equal to $P_{G}$. Additionally, it is noteworthy that an increase in the signal-to-interference-plus-noise ratio threshold ($SINR_{th}$) adds to an increase in the total system energy efficiency. Furthermore, as shown in Figure 10, the same performance is obtained for both analytical and deep learning models, indicating that raising the value of $P_{G}$ leads to a decrease in the value of the system’s achievable data rate (*R*). Also, the increase in $SINR_{th}$ values leads to a decrease in the value of R. The results shown in Figure 9 and Figure 10 show that a higher threshold ($SINR_{th}$) for the signal-to-interference-plus-noise ratio indicates that the communication signal is of higher quality when contrasted to noise and interference. The system reduces the influence of noise and interference by prioritising stronger and clearer signals by setting a higher $SINR_{th}$. This results in a more effective use of the resources and spectrum that are available, which raises the system’s total energy efficiency and achievable data rate. In essence, a greater $SINR_{th}$ contributes to increased signal quality, which in turn improves the communication system’s energy efficiency.

**Figure 11:**
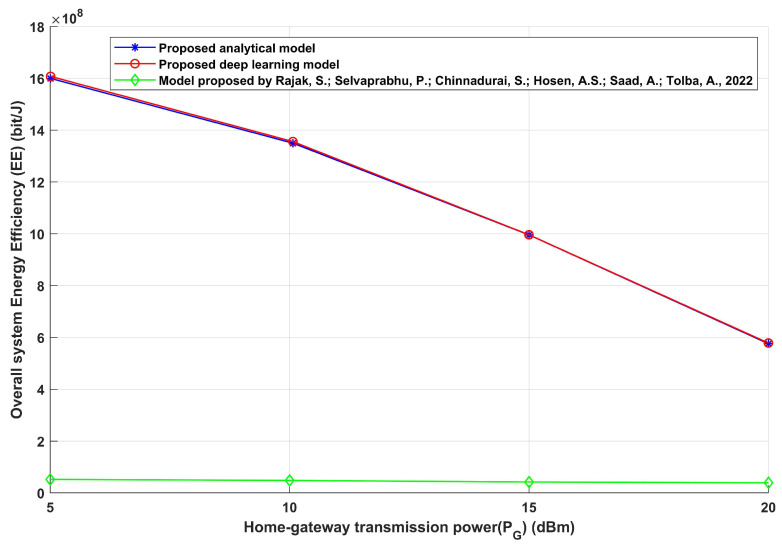
Home gateway transmission power ($P_{G}$) versus overall energy efficiency (EE) [21].

The suggested model has been compared with an established technique [21] to demonstrate its efficacy. This suggested strategy performs better, as evidenced by a comparison between the model presented in [21] and the proposed model. The comparison focuses on the relationship between necessary transmission power and overall energy efficiency, as shown in Figure 11. In terms of total energy efficiency, the suggested strategy performs better than the alternative, and this result can be linked to a number of important elements. Firstly, the suggested method probably uses complex algorithms or techniques to determine the necessary interference distance between home gateways and any interfered devices in order to maximize energy efficiency and achievable data rate. Transmission power can be used more effectively as a result of this optimization’s increased energy efficiency. Furthermore, by allowing the proposed technique to achieve the best possible trade-off between transmission distance and signal quality, this flexibility helps to achieve even more energy efficiency. Moreover, energy efficiency may be naturally increased by the fundamental design or architecture of the suggested methodology. To achieve this, state-of-the-art methods for modulation schemes, transmission protocols, and interference management could be used to create a paradigm that consumes less energy than its counterpart. In conclusion, the advanced optimization techniques, adaptability, and efficient architecture of the suggested technology can be attributed to its increased energy efficiency during transmission power changes. Furthermore, the suggested method enables home gateways to anticipate the maximum suitable interference distance based on channel conditions, providing accurate and trustworthy data reception via a deep learning model. This research also assesses the overall possible data rate and energy efficiency under various environmental circumstances. This complete study, which takes into account parameters like transmission power and needed $SINR_{th}$ values, provides insights for optimizing IoT networks for smart homes. Essentially, this work sets the groundwork for more durable and efficient smart home environments, emphasizing the accuracy of acquired data and contributing to the continued advancement of IoT technology.

Finally, the results show that the deep learning model and the proposed optimization have the adaptability and capabilities to respond to changing system environmental conditions, thereby improving the system’s performance in terms of energy efficiency and achievable data rates. Furthermore, while the suggested paradigm makes significant contributions to IoT for smart home connectivity, it is critical to recognize its limitations. One noteworthy restriction is the use of generated data instead of real-world experimentation. Simulations enable controlled testing and investigation of many scenarios, but they may not fully replicate the intricacies and nuances of real-world smart home environments. Factors such as user behaviour, environmental conditions, and hardware heterogeneity are difficult to fully recreate in simulations, thus affecting the validity and application of our findings in real-world scenarios. Furthermore, the generalizability of our findings may be constrained by certain assumptions and simplifications made throughout our modelling process. While we aim to create models that capture the core properties of smart home communication systems, these models will necessarily include simplifications and idealizations that may not fully represent the variety of real-world deployment circumstances. As a result, caution should be given when projecting our findings to diverse situations, and additional validation through empirical investigations in real-world settings is required to assure the robustness and applicability of our recommended solutions.

## 5. Conclusions and Future Work

This research looked into the essential aspect of improving communication reliability in smart homes by focusing on determining the optimal interference distance between home gateways and any interfering devices. These gateways, which serve as crucial hubs for data collection in IoT networks, are critical to guaranteeing seamless connectivity. This work focuses on the issues given by potential interference from other devices operating in the same frequency band, with the goal of determining the ideal interference distance. The suggested method addresses interference while simultaneously enhancing IoT connectivity strategies for smart homes. The strategy seeks to improve the reliability of communication between home gateways and their destinations by employing the Lagrange optimization technique and incorporating a 1 D-CNN. This optimization is critical for the effective and precise transmission of information from smart home devices. This paper’s contributions go beyond interference control. The recommended approach is to increase IoT network connections, particularly in difficult situations. The suggested model performs optimally under various environmental conditions, as demonstrated by system throughput and energy efficiency results. The interference problem has been studied and solved using deep learning and the Lagrange optimization technique. Both strategies were used to forecast the optimal interference distance in order to improve IoT communication networks. Furthermore, based on analytical and deep learning, it has been demonstrated that the interference distance must be greater than the transmission distance between any sender and receiver in order to avoid or reduce interference at any destination. To provide dependable data transmission in a variety of situations, it takes into account variables such as interference distance, path loss, signal-to-interference-plus-noise ratio ($SINR_{th}$), transmission power, and the existence of various interfering devices. For future works, we are aiming to look into using AI algorithms to analyze real-time data streams from smart home devices and sensors, allowing interference mitigation tactics to be optimized in real time. This dynamic technique has the potential to dramatically increase the robustness and efficiency of communication in smart home situations. Furthermore, looking into the potential of emerging technologies like edge computing and blockchain to supplement interference management approaches could improve the reliability and security of smart home communication systems while also opening up new opportunities for innovative applications and services.

## Figures and Tables

**Table 1:** Simulation Parameters.

Parameter	Value
*N*	−174 dBm/Hz [29]
*B*	10 Mbit/s [30]
α	4
$P_{G}$	23 dBm [31]
$P_{C}$	23 dBm [31]
$P_{D}$	23 dBm [31]
$SINR_{th}$	20 dB [31]
Pathloss between CUE and BS	$148+40\log_{2}\left(\frac{d_{CB}}{\mathrm{km}}\right)$
Pathloss between D2D link	$128.1+37.6\log_{2}\left(\frac{d_{DD}}{\mathrm{km}}\right)$

## Data Availability

Data are contained within the article.
